# Myelofibrosis at diagnosis is associated with the failure of treatment-free remission in CML patients

**DOI:** 10.3389/fphar.2023.1212392

**Published:** 2023-07-04

**Authors:** Henrike Jacobi, Margherita Vieri, Marlena Bütow, Carolina Y. Namasu, Laura Flüter, Ivan G. Costa, Tiago Maié, Katharina Lindemann-Docter, Nicolas Chatain, Fabian Beier, Michael Huber, Wolfgang Wagner, Martina Crysandt, Tim H. Brümmendorf, Mirle Schemionek

**Affiliations:** ^1^ Department of Hematology, Oncology, Hemostaseology and Stem Cell Transplantation, Faculty of Medicine, RWTH Aachen University, Aachen, Germany; ^2^ Center for Integrated Oncology Aachen Bonn Cologne Düsseldorf (CIO ABCD), Aachen, Germany; ^3^ Institute for Computational Genomics, RWTH Aachen University, Aachen, Germany; ^4^ Institute of Pathology, Faculty of Medicine, RWTH Aachen University, Aachen, Germany; ^5^ Institute of Biochemistry and Molecular Immunology, RWTH Aachen University, Aachen, Germany; ^6^ Helmholtz-Institute for Biomedical Engineering, Faculty of Medicine, RWTH Aachen University, Aachen, Germany; ^7^ Institute for Stem Cell Biology, RWTH Aachen University Medical School, Aachen, Germany

**Keywords:** CML, TFR, fibrosis, leukemic stem cells, LSC, MSc

## Abstract

The management of patients with chronic myeloid leukemia (CML) has been revolutionized by the introduction of tyrosine kinase inhibitors (TKIs), which induce deep molecular responses so that treatment can eventually be discontinued, leading to treatment-free remission (TFR) in a subset of patients. Unfortunately, leukemic stem cells (LSCs) often persist and a fraction of these can again expand in about half of patients that attempt TKI discontinuation. In this study, we show that presence of myelofibrosis (MF) at the time of diagnosis is a factor associating with TFR failure. Fibrotic transformation is governed by the action of several cytokines, and interestingly, some of them have also been described to support LSC persistence. At the cellular level, these could be produced by both malignant cells and by components of the bone marrow (BM) niche, including megakaryocytes (MKs) and mesenchymal stromal cells (MSCs). In our cohort of 57 patients, around 40% presented with MF at diagnosis and the number of blasts in the peripheral blood and BM was significantly elevated in patients with higher grade of MF. Employing a CML transgenic mouse model, we could observe higher levels of alpha-smooth muscle actin (α-SMA) in the BM when compared to control mice. Short-term treatment with the TKI nilotinib, efficiently reduced spleen weight and *BCR::ABL1* mRNA levels, while α-SMA expression was only partially reduced. Interestingly, the number of MKs was increased in the spleen of CML mice and elevated in both BM and spleen upon nilotinib treatment. Analysis of human CML-vs healthy donor (HD)-derived MSCs showed an altered expression of gene signatures reflecting fibrosis as well as hematopoietic support, thus suggesting MSCs as a potential player in these two processes. Finally, in our cohort, 12 patients qualified for TKI discontinuation, and here we observed that all patients who failed TFR had BM fibrosis at diagnosis, whereas this was only the case in 25% of patients with achieved TFR, further supporting the link between fibrosis and LSC persistence.

## 1 Introduction

Chronic myeloid leukemia (CML) is a prime example of the success of molecular-targeted therapy. The implementation of tyrosine kinase inhibitors (TKIs) that bind to and inhibit the oncogenic driver BCR::ABL1 has revolutionized treatment outcomes in this cancer entity. However, primary or secondary resistance, as well as leukemic stem cell (LSC) persistence, remain a problem that hinders the cure of this disease. The persistence of LSCs is clinically reflected by residual *BCR::ABL1*-expression in CML patients in major or deep molecular response (MMR, DMR), which often persist at a very low level despite long-term treatment. LSC persistence is a major obstacle for one of the main goals of CML therapy, i.e., treatment-free remission (TFR), where patients remain in MMR or DMR after discontinuation of TKI therapy. The first large clinical trial on TFR has shown that about half of CML patients relapse, usually within the first 6 months (Mahon et al., 2010). Further studies focused on additional aspects such as the specific TKI, course of initial response, duration or level of DMR prior to discontinuation of therapy with slight deviations in outcome [reviewed in (Cortes et al., 2019; Saglio and Gale, 2020; Osman and Deininger, 2021)]. Some predictive factors for success of TFR have been proposed, such as a longer period of DMR before discontinuation, older age, lower SOKAL risk score or longer time of TKI treatment before discontinuation [reviewed in (Atallah and Sweet, 2021)]. Based on the large number of studies, it is currently calculated that about two-thirds of newly diagnosed CML patients are eligible for TKI discontinuation, with half of them remaining in TFR.

A variety of mechanisms that could be responsible for the persistence of LSCs have been described. Looking at the microenvironment, we now know that the inflammatory signature of LSCs is driven not only by autocrine but also by paracrine mechanisms (Kuepper et al., 2019). Interestingly, some of the cytokines that have been shown to promote LSC persistence, such as interleukin-6 (IL-6) (Welner et al., 2015; Kuepper et al., 2019), interleukin-1β (IL-1β) (Zhang et al., 2016), tumor necrosis factor (TNF) (Herrmann et al., 2019; Agarwal et al., 2021) or transforming growth factor-beta (TGF-β) (Naka et al., 2010), have also been shown to support fibrotic processes in the bone marrow (BM).

BM fibrosis or myelofibrosis (MF) is a complex process that leads to the deposition of excessive extracellular matrix (ECM) within the BM, which, upon progression slowly replaces hematopoietic cells. The development of MF is potentially possible in all myeloid neoplasms (Sabattini et al., 2010) and is typically a slow and gradual process, where rapid changes of the ECM are rare. The origin of BM fibrosis is still poorly understood, but it is thought to involve the dysregulation of multiple cellular pathways, including cytokine signaling and inflammatory responses [reviewed in Gleitz and Benabid et al. (Gleitz et al., 2021)]. In the BM of fibrotic Philadelphia-negative (Ph^−^) myeloproliferative neoplasms (MPNs), Gli^+^ and LepR1^+^ MSCs are driven into differentiation by a proinflammatory environment into a pro-fibrotic, myofibroblast-like cell type that then deposits excessive reticulin and collagen fibers (Ignotz et al., 1987; Lataillade et al., 2008; Decker et al., 2017; Schneider et al., 2017). The severity of BM fibrosis is often described with MF grades ranging from lower prefibrotic grade 1 to defined MF with grades 2–3 (Thiele et al., 2005; Swerdlow et al., 2017). In CML, the proportion of patients with MF varies widely from 30% to 100% of patients (Gralnick et al., 1971; Kvasnicka et al., 2001; Buesche et al., 2003; Hamid et al., 2019). Interestingly, imatinib seems to be able to either slow down or reverse MF (Beham-Schmid et al., 2002; Bueso-Ramos et al., 2004; Thiele et al., 2004; Buesche et al., 2007). An open question is whether the degree of MF at diagnosis has an impact on response to TKI treatment, with different studies showing conflicting results (Kantarjian et al., 2005; Buesche et al., 2007; Tanrikulu Simsek et al., 2016). Importantly, Buesche et al. (2007) showed that MF before start or within the first 3 months of imatinib therapy did not influence the probability of achieving a complete hematologic response during imatinib treatment. However, deeper responses such as complete cytogenetic remissions or MMR were less likely in patients with MF compared to patients without MF.

Given the supposed impact of MF on TKI response and the overlapping spectrum of cytokines that influence this process as well as LSC persistence, we analyzed here the relationship between BM fibrosis and TFR outcome in a small cohort of patients.

## 2 Materials and methods

### 2.1 Statistical analysis of patient data

Clinical data were collected from 57 patients from the RWTH Aachen University Hospital. All patients provided informed consent and analysis of human samples was approved by the local ethics board of the medical faculty RWTH Aachen (EK 206/09 and EK 391/20). MF grades were assessed by the local pathology department of the RWTH Aachen University Hospital for all patients except 3. Median age ± standard deviation of patients was 54.7 ± 15.4 years, 42 (74%) were male. Correlations between MF grade at diagnosis and TFR were assessed for patients undergoing discontinuation of the TKI after being at least 1.5 years in deep molecular remission (MR4 (Cross et al., 2012), *BCR::ABL1* <0.01%). Success of discontinuation was defined as stable MMR for at least 1 year without need for re-treatment. TFR failure was defined as loss of MMR. Optimal response (OR) at 12 months was achieved if the patient’s *BCR::ABL1* levels were below 0.1% (MMR as defined in the International Randomized Study of Interferon and STI571 trial [IRIS] (Hughes et al., 2010)) 12 months after diagnosis and start of TKI treatment.

### 2.2 Animal experiments

FVB/N *SCLtTA/BCR::ABL1* (double transgenic, CML) mice and FVB/N *SCLtTA* (single transgenic, control) siblings were bred in-house and tetracycline was added to the drinking water (0.5 g/L, Sigma-Aldrich, St. Louis, MO, United States) in order to avoid the expression of *BCR::ABL1* (Institute for Laboratory Animal Science, Uniklinik RWTH Aachen). BM cells of control or CML mice were isolated from femur and tibia, separated by cell straining and 2 × 10^6^ cells were transplanted into the wildtype 10 Gy irradiated FVB/N CD45.1^+^ recipient mice by tail vain injection (Janvier Labs^®^). Mice were treated with cotrimoxazole (Ratiopharm, Ulm, Germany) for 2 weeks after transplantation. One week after transplantation, *BCR::ABL1* expression was induced by withdrawing tetracycline. After an additional week, treatment of CML mice with nilotinib (NIL, 50 mg/kg body weight) by daily gavage over 3 weeks was started. NIL (MedChemExpress, Monmouth Junction, NJ, United States) was resolved in 10% N-Methyl-2-pyrrolidon (Carl Roth) and 90% polyethylene glycol 300 (Sigma-Aldrich). Treatment controls were performed on both control and CML mice by administering the vehicle following the same procedure. All animal experiments were approved by the local authorities of North Rhine-Westphalia, Germany (LANUV Az. 84–02.04.2013.A072).

### 2.3 Immunohistochemistry (IHC) staining

Formalin preserved sternum specimen were embedded in paraffin and sectioned with a microtome and further processed following manufacturer instructions (Cell Signaling^®^). To perform the staining, sections were de-paraffinized and hydrated, followed by antigen retrieval with citrate buffer. Sections were then blocked by peroxidase followed by serum blocking. Sections were incubated overnight at 4°C with an anti-alpha-smooth muscle actin (α-SMA) primary antibody (add antibody number) at a 1:800 dilution. Next, slides were washed and incubated for 30 min with the secondary antibody (HRP, Rabbit #8114; Cell Signaling Technology^®^). Finally, sections were developed with diaminobenzidine, counterstained with hematoxylin and mounted with coverslips. Quantification of IHC staining was carried out using Fiji software (ImageJ^®^), after performing a color deconvolution (H DAB) (Crowe and Yue, 2023).

### 2.4 Fibrosis staining/grading of CML patients

After Formalin preservation, decalcification (EDTA) and paraffin embedding, 2.5–4 µm slides were produced by a microtome. For staining, a silver impregnation technique was used. The sections were de-paraffinized and hydrated and workflow was performed according to the following protocol: potassium permanganate (1%) for 5 min, oxalid acid (2%) for 5 min, iron alum for 5 min, silver solution ammoniacal for 10 s, formalin (5%) for 3 min, sodium thiosulphate (2%) for 2 min. Between each step, washing with distilled water was performed. Afterwards, nuclear fast red solution was used for 10 min for counterstaining. After immersing in alcohol (ascending, 70%–100%) and xylol, the slides were mounted with coverslips.

Grading occurred according to the WHO grading system (semiquantitative bone marrow fibrosis (MF) grading system proposed by WHO Classification of Tumours of Hematopoetic and Lymphoid Tissues (Swerdlow et al., 2017)). The four grades (MF-0 to MF-3) are defined as follows: MF-0: Scattered linear reticulin with no intersections, MF-1: Loose network of reticulin with many intersections, especially in perivascular areas, MF-2: Diffuse and dense increase in reticulin with extensive intersections, occasionally with focal bundles of thick fibers mostly consistent with collagen and/or associated with focal osteosclerosis, MF-3: Diffuse and dense increase of reticulin with extensive intersections and coarse bundles of thick fibers, consistent with collagen, usually associated with osteosclerosis. In heterogenous patterns, the final grade is determined by the highest grade present in at least 30% of the marrow area.

### 2.5 Flow cytometry analysis of murine bone marrow and spleen cells

BM and spleen cells were harvested and lysis of red blood cells was performed with a ammonium-chloride-potassium solution (AKC). Subsequently, cells were washed with PBS (Pan Biotech^®^) + 2% fetal bovine serum (FBS, PAN Biotech^®^) and then incubated for 20 min at 4°C with a 1:100 dilution with anti-CD41 conjugated with APC Cy7 fluorochrome (BioLegend^®^) in the dark. Next, cells were washed and resuspended in PBS + 2% FBS. In addition, FACS staining of B-cells (B220^+^), granulocytes (GR-1^+^/CD11b^+^), T-cells (CD3^+^), blasts (KIT^+^), and erythrocytes (Ter119^+^) was performed. Flow cytometer measurements were carried out using Gallios flow cytometer (Beckman Coulter^®^) and analyzed with Kaluza analysis software (Beckman Coulter^®^).

### 2.6 Isolation of MSCs

Human MSCs were obtained from BM aspirates of five CML patients at time of diagnosis and from hip bone donated after hip replacement surgeries of five healthy donors (HD). Cells were isolated by tissue culture plastic adherence after the mononuclear cell fraction was separated by Ficoll. Cells were seeded at 160,000 cells per cm^2^ in Dulbecco’s Modified Eagle Medium (DMEM) low glucose (Gibco^®^) supplemented with 10% of FBS, 1% each of L-glutamine, penicillin/streptomycin (both Gibco^®^) and cultured at 37°C with 5% CO_2_. After 24 h, the medium was replaced and non-adherent cells removed by washing with phosphate-buffered saline (PBS, PAN Biotech^®^). Afterwards, adherent cells were maintained with medium replacement twice a week, detached with 0.25% trypsin-EDTA (Gibco^®^) after reaching confluence. MSCs were used for our experiments at passage 2 (p2).

### 2.7 Trilineage differentiation assays

MSCs were tested for their ability to differentiate *in vitro* into adipocytes, osteocytes and chondrocytes (Sudo et al., 2007). Briefly, in order to perform adipogenic differentiation, cells were cultured in medium consisting of DMEM high glucose (Gibco^®^), 10% FBS, 1 µM dexamethasone, 200 µM indomethacin, 500 µM 3-isobutyl-1-methylxantine and 10 μg/mL insulin (all Sigma^®^). On day 14–21, adipogenic cultures were washed with PBS, fixed in PFA 4% and stained with Oil Red O (Sigma^®^). For the osteogenic differentiation, cells were cultured in medium consisting of DMEM high glucose (Gibco^®^) supplemented with 10% of FBS (PAN Biotech^®^), 1% L-glutamine, 1% penicillin/streptomycin (both Gibco^®^), 10 nM dexamethasone, 10 mM β-glycerolphosphate and 50 µM ascorbic acid (Sigma^®^). After 21 days, cells were washed with PBS, fixed in PFA 4% and stained with Alizarin Red (Sigma^®^). For the chondrogenic differentiation, cells were cultured in medium consisting of DMEM high glucose (Gibco^®^), 1% L-glutamine, 1% penicillin/streptomycin (both Gibco^®^), 100 nM dexamethasone, 0.17 mM L-Ascorbic acid-2-PO4, 100 μg/mL sodium pyruvate, Insulin Transferrin Selenium supplement (ITS, Sigma^®^) and 10 ng/mL TGF-β. On day 14, cultures were washed with PBS, fixed in PFA 4% and stained with Alcian blue (Sigma^®^). After visual inspection and image acquisition, quantification of the three stainings was performed using the following solvents: isopropanol for adipogenic staining, 10%-cetylpyridinium chloride solution for osteogenic staining and 6M Guanidine HCl solution for chondrogenic staining, the relative absorbance was measured with a plate reader (Multiskan FC, Thermo Fisher^®^).

### 2.8 MSC phenotype

MSC phenotype was assessed at passage 2 (p2) using the following monoclonal antibodies: anti-CD73-FITC (eBioscience^®^); anti-CD34-FITC (BD Biosciences^®^, BD); anti-CD45-PE (BD^®^); anti-CD90-PerCP-Cy5.5 (eBioscience^®^); anti-CD13-PE (eBioscience^®^); anti-CD11b -FITC (BD^®^) anti-CD44-APC and anti-HLA-DR-FITC (BD^®^). Staining of at least 50,000 cells was performed at 4°C for 15 min in the dark in presence of 1% human serum to avoid unspecific binding. Samples were acquired with a Gallios flow cytometer (Beckman Coulter^®^) and analyses were performed using FlowJo Software (BD^®^).

### 2.9 CFU-F assay

Log serial dilutions of the MSC at p2 were placed into 10 to 20 replicates in 96-well plates in the presence of MesenPure (STEMCELL Technologies) and 0.5% penicillin-streptomycin. After 14 days, colony-forming unit-fibroblast (CFU-F) were performed using MesenCult Expansion Kit (STEMCELL Technologies^®^), where the wells were stained with Giemsa (Merck) according to manufacturer’s instruction. Colonies were then counted manually. The frequency of progenitor cells in the undiluted starting population was calculated manually.

### 2.10 Microarray analysis

RNA of four HD- and four CML-derived MSCs was isolated using TRIzol™ Reagent (Thermo Fisher^®^) as previously described. RNA was hybridized on Human Clariom D Assay gene chip (Affymetrix^®^). Further steps, such as sample labeling, hybridization, washing and scanning were performed according to manufacturer’s protocols. Downstream analysis was performed in R (v4.2.2), using packages oligo (v1.62.2), Biobase (v2.58), affycoretools (v1,70), limma (v3.54.2), PCAtools (v2.10), progeny (v1.20), msigdbr (v7.5.1) and clusterProfiler (v4.6) for analysis; data.table (v1.14.8), tibble (v3.2.1) and dplyr (v1.1.1) for data wrangling; and ggplot2 (v3.4.1), ggrepel (v0.9.2), ComplexHeatmap (v2.14), patchwork (v1.1.2) and cowplot (v1.1.1) for visualization.

Robust Multichip Average preprocessing was performed. This included background subtraction, quantile normalization and summarization. The latter step was performed at the probeset level. Principal Component Analysis was performed to evaluate if each sample group was clustering together (Supplementary Figure S3B). Samples CML2 and HD4 were deemed unfit for this analysis. CML2 was treated with imatinib which could be a confounding factor. HD4 was clustering apart from the other HDs and therefore considered an outlier. Non-mapping probes, probes mapping to sexual chromosomes and probes with missing data were filtered out. A PCA was again performed after the filtering (Supplementary Figure S3C).

Differential expression analysis was performed with limma-trend with an exclusion filter for probes with a mean expression below 5 on the log2 scale, note that this still retains 439,961 probes. A linear model for the CML vs. HD comparison was fit and its statistics were estimated with empirical Bayes moderation. *p*-values were corrected for multiple testing with the Benjamini–Hochberg procedure.

PROGENy was performed on the same data fed to the differential expression analysis and setting the “top” parameter to 1,000 and “scale” to false. PROGENy results were then scaled in regard to the HD samples. Over-Representation Analysis (ORA) and Gene Set Expression Analysis (GSEA) were performed on the results from the differential expression analysis. For ORA, the background probes considered were the same as the ones present in the differential expression analysis. Only enrichment for under-expressed probes was performed as no probe passed the over-expression significance threshold during differential expression analysis. For ORA and GSEA, *p*-values were corrected for multiple testing with the Benjamini–Hochberg procedure. Chosen custom gene sets and MSigDB gensets were evaluated separately. For PROGENy and GSEA, probe information was summarized down by mean to the gene level.

### 2.11 Statistical analyses

1-way or 2-way analysis of variance (ANOVA) were applied for multiple comparison analysis, with Tukey’s or Bonferroni’s post-hoc-test. Unpaired *t*-test was used to compare the means of two groups. Fisher’s exact test was used for Contingency Table. Statistical calculations were performed with GraphPad Prism (GraphPad Software, La Jolla, CA, United States). Data are presented as mean ± standard deviation (SD). *p < 0.05, **p < 0.01, ***p < 0.001, ****p < 0.0001 were considered statistically significant.

## 3 Results

### 3.1 Clinical characteristics of CML patients

**FIGURE 1:**
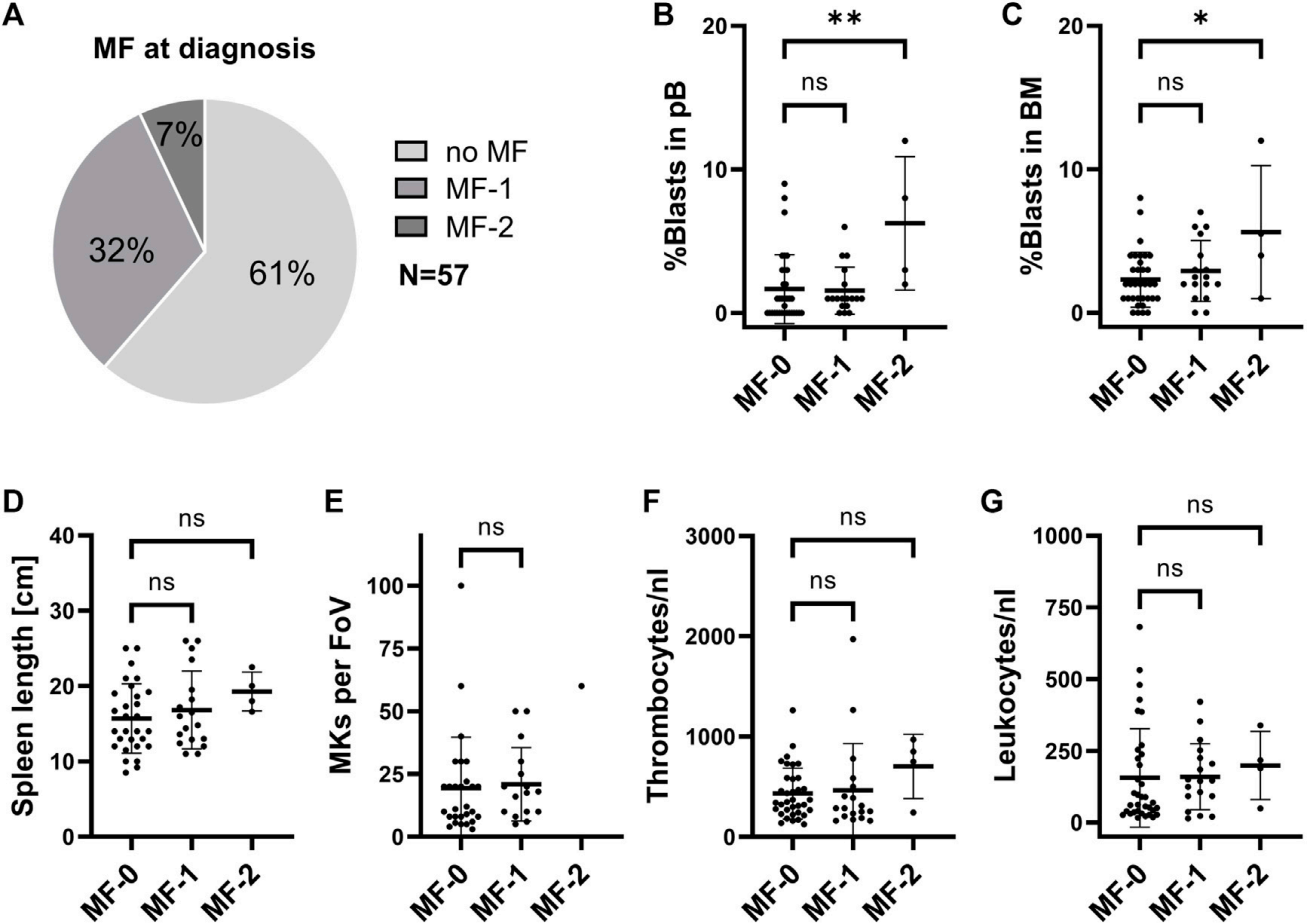
Clinical parameters in CML patients at initial diagnosis with different stages of BM fibrosis. **(A)** Proportion of CML patients with myelofibrosis (MF) in our cohort. **(B)** Peripheral blood (pB) blast cell count, **(C)** bone marrow (BM) blast cell count, **(D)** spleen length, **(E)** BM megakaryocytes (MKs), **(F)** pB thrombocytes and **(G)** pB leukocytes were assessed by the time of initial diagnoses and are represented, depending on the stage of MF. FoV: field of view.

Based on the conflicting reports on the frequency of MF, we first assessed the proportion of patients with MF at diagnosis in our CML cohort (n = 57). Here, 38.6% (n = 22) had MF at the time of diagnosis, with 31.6% having MF stage 1 (n = 18) and 7.0% (n = 4) having stage 2 (Figure 1A). No grade 3 MF was observed. As MF has previously been suggested to be an adverse prognostic marker in CML (Buesche et al., 2003; Buesche et al., 2004; Buesche et al., 2007) we started to compare different clinical parameters among patients with different stages of MF. Here we observed that the percentage of peripheral blood (pB) and BM blasts is significantly higher in CML patients with MF grade 2 (Figures 1B,C). Spleen length, the number of BM megakaryocytes (MKs), platelet counts and leukocyte counts did not significantly change upon MF development (Figures 1D–G). In our cohort, presence of MF also does not correlate with response to treatment at 12 months (Supplementary Figure S1A), risk scores (Supplementary Figure S1B–D) or patient age at diagnosis (Supplementary Figure S1E). Furthermore, TFR outcome also did not correlate with common risk scores (Supplementary Figure S1F–H).

### 3.2 Increase in megakaryocytes in α-SMA positive murine CML bone marrow upon TKI treatment

**FIGURE 2:**
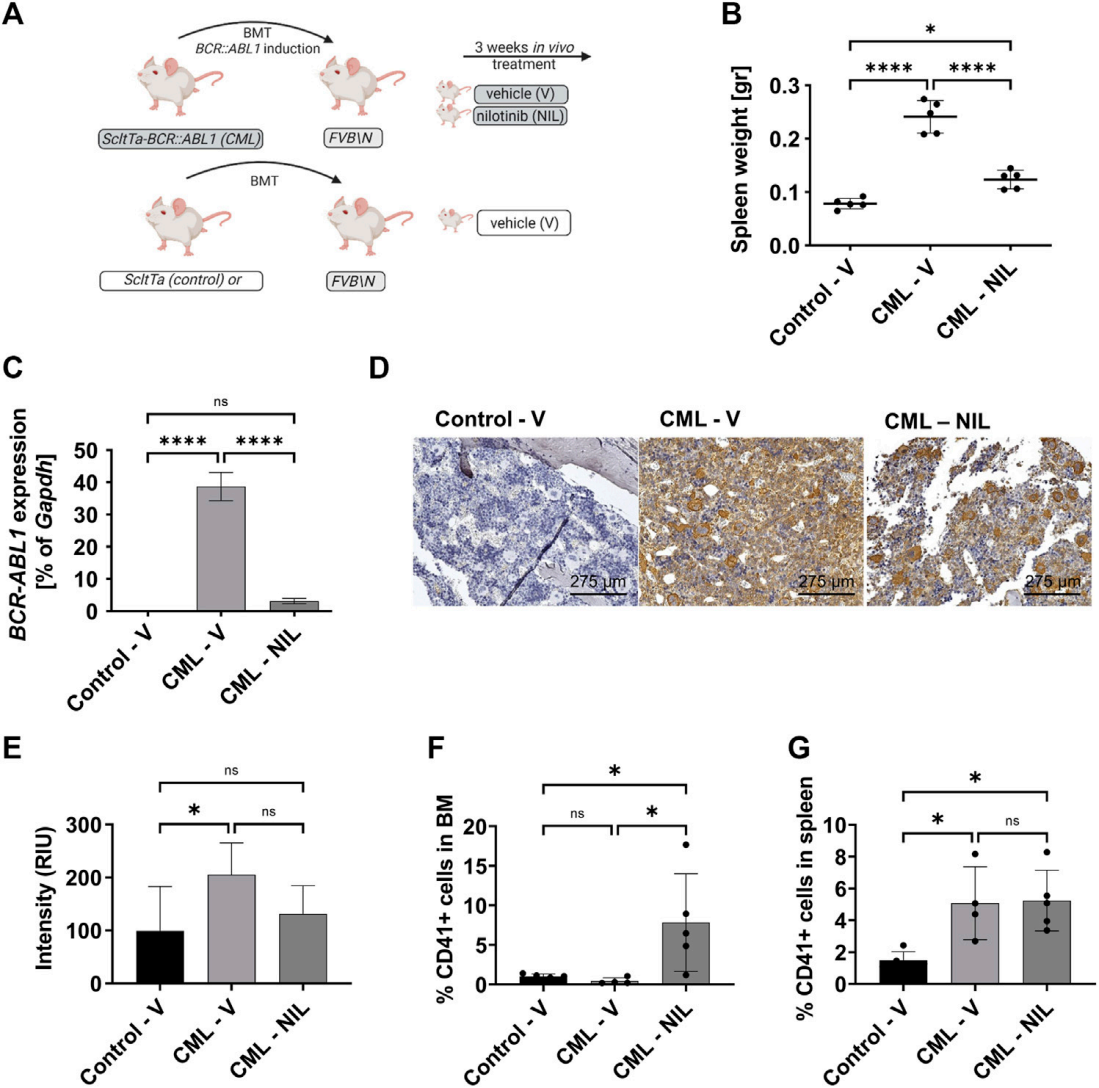
CML transgenic mice show increased megakaryocytes upon TKI treatment. **(A)** Schematic representation of the mouse model and experimental set up. **(B)** Spleen weight at endpoint analysis. **(C)** *BCR::ABL1* mRNA expression. Gapdh was used as housekeeping gene. **(D)** α-SMA staining of the sternum showing a representative example of each group. **(E)** Quantification of α-SMA staining. **(F)** FACS-immunophenotype of CD41^+^ megakaryocytes in the bone marrow. **(G)** FACS-immunophenotype of CD41^+^ megakaryocytes in the spleen. V: Vehicle; NIL: nilotinib; RIU: relative intensity unit. *p < 0.05, ****p < 0.0001.

Using the transgenic CML model we previously confirmed that LSCs persist despite BCR::ABL1 inhibition (Schemionek et al., 2010; Hamilton et al., 2012). Further studies have revealed that CML development induces expansion of osteoblastic lineage cells and MF has been suggested to occur in these mice as analyzed by collagen (Schepers et al., 2013) or reticulin-staining (Reynaud et al., 2011). As the grade of fibrosis is usually low in CML, we here used α-SMA staining in order to dissect early fibrotic changes. To exclude any possibility of transgene expression in the non-hematopoietic compartment we first transplanted 2 × 10^6^ BM cells, isolated from either *SCLtTA*/*BCR::ABL1* CML or *SCLtTA* control mice into 10 Gy irradiated recipients and allowed these cells to engraft for 1 week before inducing *BCR::ABL1* expression. 2 weeks after induction of the oncogene, mice were treated with either nilotinib (NIL) or vehicle (V) control for 3 weeks (Figure 2A). Upon autopsy the increase in spleen weight was evident in the CML V group compared to the control V group. This was largely reverted upon NIL treatment as expected and previously shown (Figure 2B). In line with these data, expression of *BCR::ABL1* was evident in V-treated CML mice but significantly decreased upon NIL treatment (Figure 2C). A detailed FACS immunophenotyping revealed that the B-cells were driving the disease phenotype and responded to TKI treatment (Supplementary Figure S2), as we had previously observed (Butow et al., 2023). Sternum sections of the BM were stained for α-SMA that is known to be expressed in myofibroblasts. Indeed, we observed a significant increase in V-treated CML mice that was reverted to some extend upon NIL treatment (Figures 2D,E). To our surprise, α-SMA appeared to be also expressed in CML MKs, which are a supposed driver of fibrotic transformation in MPN. FACS analysis revealed that MK percentages were not altered in the BM but increased in the spleen of CML transgenic mice and interestingly elevated in both organs upon TKI treatment (Figures 2F,G).

### 3.3 Phenotypic profiling of CML MSCs

**FIGURE 3:**
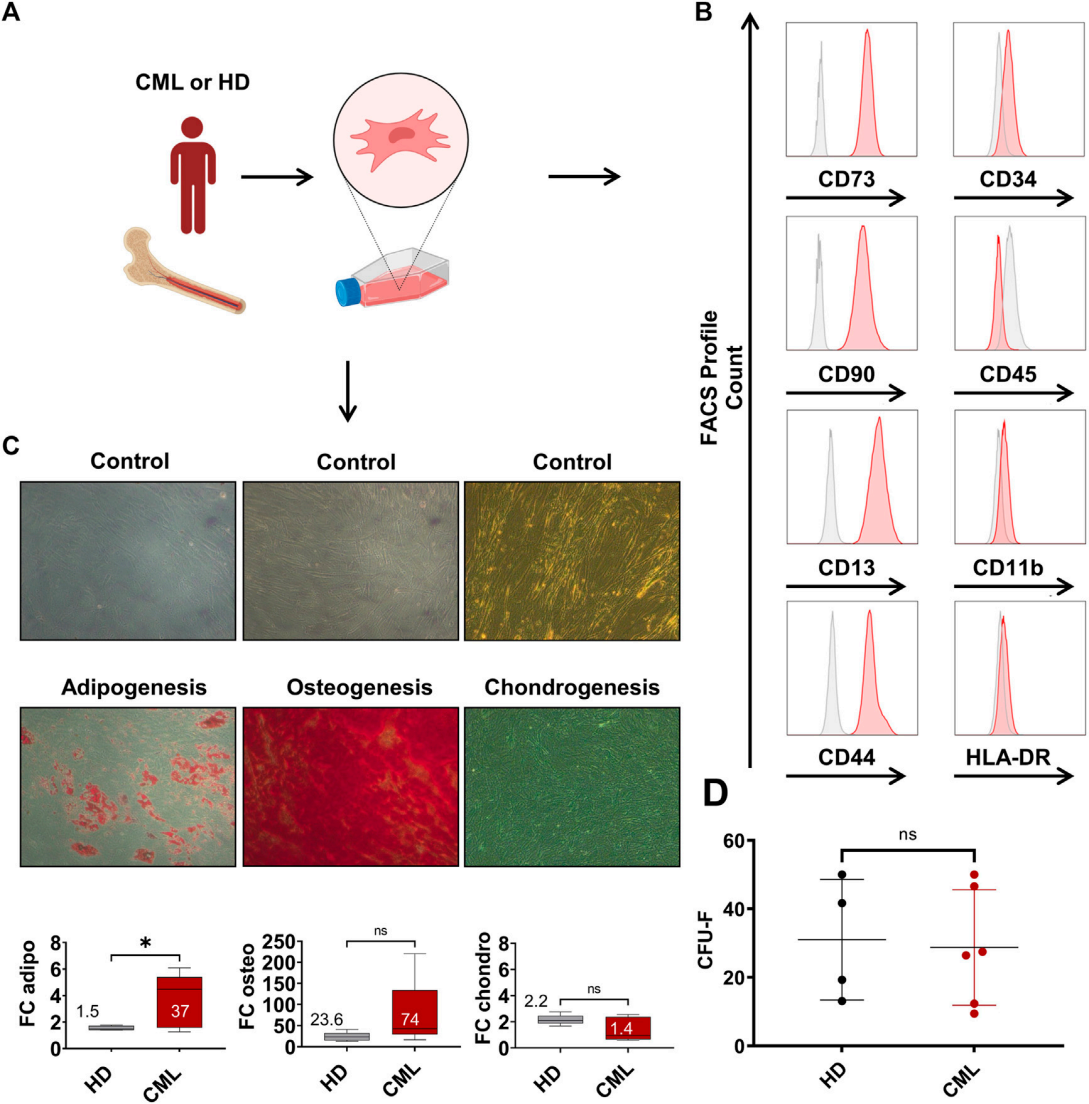
Characterization of CML MSCs shows increased differentiation, but unaltered clonogenic potential. **(A)** MSCs were extracted from the BM and subsequently isolated via plastic adherence. **(B)** MSC FACS profile of MSCs in p2. **(C)** Healthy donor- (HD) CML-derived MSCs were analyzed for their potential to differentiate towards the adipogenic, osteogenic, or chondrogenic lineage (n = 5/5). Fold change (FC) over control of the absorbance is reported in the box plot at the bottom of panel **(C)**. Mean FC is indicated in the graph. **(D)** CFU-F of HD-vs CML-derived MSC. *p < 0.05.

Mesenchymal stromal cells (MSCs) were either isolated from BM of CML patients or the hip bone of HDs via plastic adherence (Figure 3A) and their immunophenotype was analyzed at p2 (Figure 3B). The FACS analyses showed the expression of common MSC markers, such as CD73, CD90, CD14 and CD44. Typical hematopoietic markers, such as CD34, CD45, CD11b and HLA-DR were not present, consistent with the characteristic pattern of mesenchymal surface markers. Next, we evaluated their differentiation potential into the adipogenic, osteogenic, and chondrogenic lineage (Figure 3C). The adipogenic potential of CML-derived MSCs was significantly enhanced compared to HD. The osteogenic differentiation of CML-derived MSCs appeared to be elevated but that was rather due to a single CML sample showing an outstanding osteogenic capacity. Chondrogenic differentiation did not differ in CML vs. HD-derived samples. In order to evaluate the clonogenic capacity of CML MSCs, we performed a limiting dilution CFU-F assays (Figure 3D). These data show that there was no difference observed in CML-vs HD-derived MSCs. Overall, although CML-derived MSCs showed an increased capacity to differentiate into the adipogenic lineage, no other substantial phenotypic differences were observed in the two groups.

### 3.4 CML MSCs are driven towards a fibrotic and potentially LSC-supporting expression profile

**FIGURE 4:**
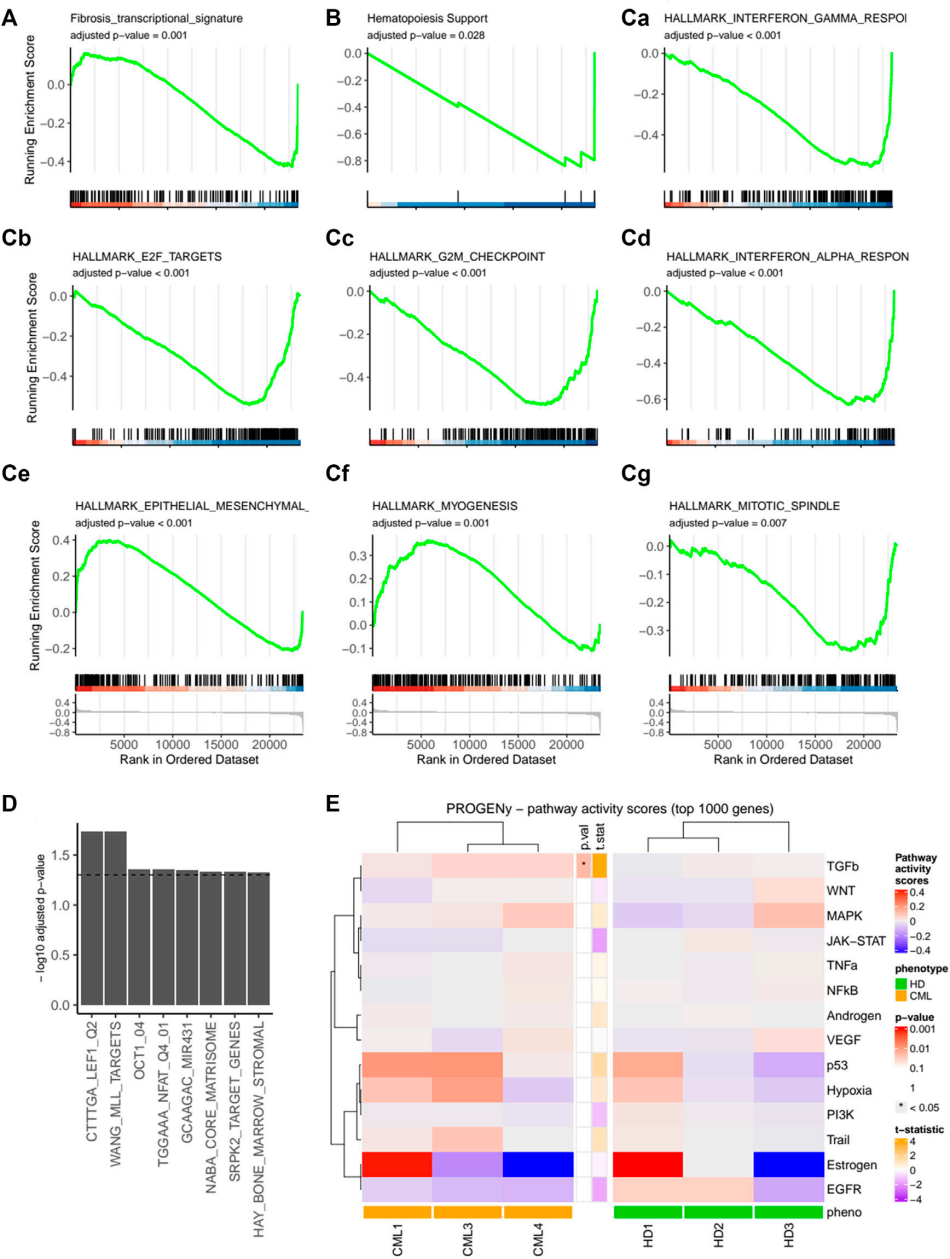
Expression profile of the mesenchymal CML fibrotic niche. HD-MSCs or CML-MSCs were expanded until passage 2 and RNA expression analysis was performed using a microarray. GSEA of custom gene sets for fibrosis **(A)** and hematopoiesis support **(B)** in CML vs. HD are shown. In **(C)**, hallmark gene sets with adjusted *p*-value < 0.05 are shown. Specifically, Ca shows Interferon gamma response, Cb E2F targets, Cc G2M checkpoint, Cd Interferon alpha response, Ce epithelial-to-mesenchymal transition, Cf myogenesis, Cg mitotic spindle. **(D)** Selection of significant (adjusted *p*-value < 0.05) Molecular Signatures Database (MsigDB) gene sets for over-representation analysis of downregulated genes in CML vs. HD. *p < 0.05. **(E)** Pathway responsive genes (PROGENy) of multiple comparisons of HD-MSCs or CML-MSCs. Upregulated pathways are shown in red and downregulated ones in blue.

To determine the gene expression signature of CML-vs HD-MSCs, we performed a microarray analysis. Overall changes where rather mild in these MSCs that were expanded until p2, as shown by the volcano plot (Supplementary Figure S3A). However, initial quality control showed specific clustering of CML vs. HD samples and revealed HD 4 as an outlier (Supplementary Figure S3B), potentially due to technical reasons and was therefore excluded from subsequent analysis. In addition, CML 2 was previously treated and therefore likewise excluded from further analyses (Supplementary Figure S3C). We could observe significant changes in a defined fibrotic gene signature set, which was previously derived from the analysis of expression profiling datasets of human organs with fibrosis-related diseases (Wang et al., 2020) (Figure 4A). Furthermore, an altered expression profile was also observed in a defined gene set for hematopoiesis support (Figure 4B). Next, gene set expression analysis (GSEA) was performed using the molecular signature database (MSigDB) hallmark gene sets collection (Figure 4C). A significant alteration of signaling pathways involved in inflammation was found, such as interferon gamma and alpha response. Interestingly, a significant upregulation of epithelial-to-mesenchymal transition (EMT) signature was also observed. Among the downregulated pathways (Figure 4D), another gene set of high interest that was significantly downregulated was the one containing genes with at least one occurrence of the motif detected by MIR431 (Jiang et al., 2018). This microRNA has been previously found downregulated in AML and its overexpression induced downregulation of TGF-β as well as epithelial-to-mesenchymal transition-related genes. Finally, we show 14 additional signatures of pathway perturbation (pathway responsive genes, PROGENy analysis) (Schubert et al., 2018) in our two cohorts and found a significant upregulation of the TGF-β pathway in the CML-derived MSCs (Figure 4E).

### 3.5 TFR often fails in patients with MF at diagnosis

**FIGURE 5:**
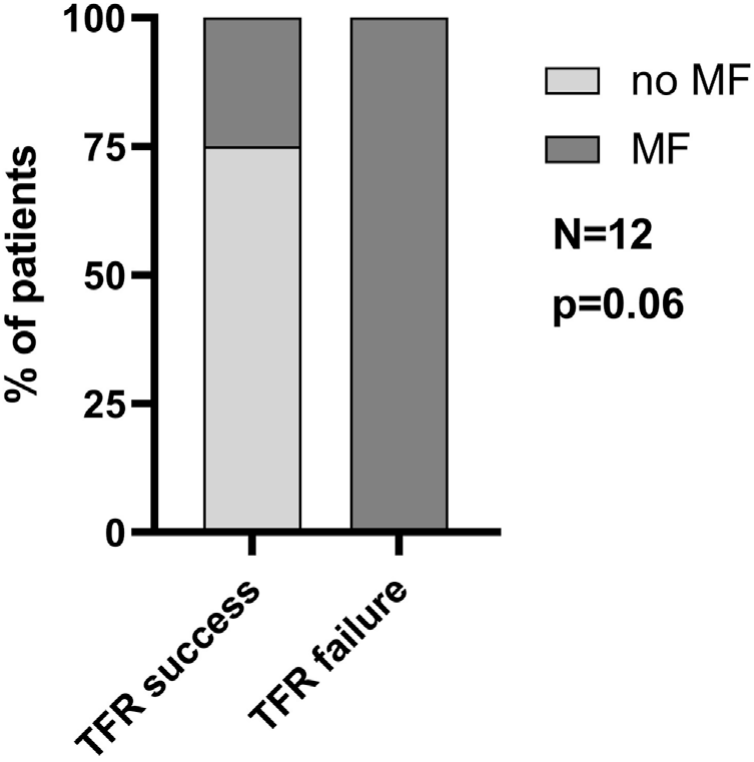
Presence of myelofibrosis at initial diagnosis is associated with TFR failure. The percentage of patients with or without MF that successfully reached TFR. N = 12.

The success of TFR is compromised by the persistence of LSCs in CML patients on TKI therapy, which re-expand upon TKI discontinuation. Based on the expression analysis of primary patient-derived MSCs indicating LSC-supporting potential, we investigated the impact of MF on TFR. A minority of CML patients underwent TKI discontinuation. In our cohort of patients with available MF grading at diagnosis, 12 patients discontinued TKI therapy after at least 1.5 years of MR4 or better. The clinical parameters of these patients are described in Table 1 and Supplementary Table S1. In the small cohort of patients, no significant differences in age, treatment duration or duration of MR4/5 were observed when comparing patients who succeeded or failed TFR. Of those 12 patients, 4 failed TFR (33%) and interestingly, all presented with MF at diagnosis (Figure 5). There was one additional patient with MF grade 1 at diagnosis who failed TFR, but he was excluded from this analysis, as he only reached MR3 for 16 months and therefore did not fulfil the current criteria for TKI discontinuation. Out of 8 patients who achieved TFR, 2 (25%) had MF grade 1 at diagnosis, whereas the other 6 (75%) had no BM fibrosis. Notably, all patients without MF at diagnosis achieved TFR, while all patients with MF grade 2 at diagnosis, failed. Only patients with MF grade 1 varied in TFR success, half of them failing, the other succeeding (Table 1).

**TABLE 1:** Characteristics of patients that discontinued TKI.

No.	Age	Sex	MF grade	TFR success	TKI	Years of TKI	Years of MR4-MR5	Years of TFR	Study
1	57	male	0	Yes	Nilotinib	3	2.75	8.5	EnestFreedom
2	39	male	0	Yes	Nilotinib	5	>2	6.5	EnestFreedom
3	48	male	0	Yes	Imatinib	12	9	2	-
4	64	female	0	Yes	Nilotinib	8	3.5	1.25	-
5	58	female	0	Yes	Bosutinib	5	3.5	4	Endure
6	21*	female	0	Yes	Nilotinib	(3 &) 6	2.5	5	(EnestFreedom) Endure
7	58*	male	1	No	(Imatinib) Nilotinib	(3 &) 8	4	-	(EuroSki) NAUT
8	59	male	1	Yes	Nilotinib	3	2	6.25	CML-V/TIGER
9	57	female	1	No	Imatinib	8	5	-	-
10	57	female	1	Yes	Nilotinib	4.5	3.5	6	-
11	60**	male	1	No	Bosutinib	2.5	16 months MR3	-	-
12	74	male	2	No	Imatinib	4	3	-	-
13	60	female	2	No	Nilotinib	4	1.5	-	CML-V/TIGER

*Patients attempted discontinuation of tyrosine kinase inhibitor (TKI) two times at different timepoints as indicated and did not achieve therapy free remission (TFR) the first time.

**Patient only reached MR3 before TKI discontinuation and was not included in final analysis.

No.: number, MF: myelofibrosis. Age, sex and MF grade are given for the timepoint of diagnosis, the other parameters for the timepoint of TKI discontinuation. TFR success was evaluated as described in Material and Methods.

## 4 Discussion

In this study, we suggest a potential link between the presence of BM fibrosis in CML patients at diagnosis and persistence of LSCs that are capable to re-expand upon TKI discontinuation, thereby resulting clinically in TFR failure.

First, we could observe that in our cohort around 40% of the patients presented with MF at diagnosis and MF grade 2 correlated with higher blast counts in the blood and BM. Although the impact of BM fibrosis on prognosis was considered adverse in the pre-TKI era (Lazzarino et al., 1986; Kvasnicka et al., 2001; Buesche et al., 2003), it seems to be less relevant nowadays, due to the enormous success of TKIs and the drastic improvements in patient management that were introduced (Kantarjian et al., 2005; Tanrikulu Simsek et al., 2016). BM fibrosis at diagnosis has been shown to revert in the majority of patients upon TKI treatment (Buesche et al., 2007; Eliacik et al., 2015; Narang et al., 2017) and we likewise observed this in patient 11. Tanrikulu Simsek et al. (2016) furthermore confirmed a decrease in fibrosis grades after long-time imatinib treatment. It has to be noted that in a few single patients imatinib treatment was associated with induction of BM fibrosis (Buesche et al., 2007; Shanmuganathan et al., 2019) and interestingly, in patient 3 of our cohort we see development of low-grade fibrosis after 5.5 years of imatinib treatment. A potential mechanism of the antifibrotic properties of TKIs involves targeting of the platelet-derived growth factor receptor protein kinase α and β (Hantschel et al., 2008), which are both involved in the process of fibrogenesis. Moreover, TKIs bind and inhibit ABL itself, which is part of the signaling cascade of TGF-β (Daniels et al., 2004), another important mediator of fibrosis development. However, to our knowledge, the origin of fibrosis in CML has not been addressed so far.

In Ph^−^ MPNs, MKs and MSCs are known to play key roles in the development of fibrosis (Malara et al., 2014; Decker et al., 2017; Schneider et al., 2017; Kramann and Schneider, 2018). In the BM of our ScltTA-*BCR::ABL1* mice, high levels of α-SMA were detected, which is an early marker of fibrogenic activity (Bochaton-Piallat et al., 2016). This expression was partly reverted by nilotinib treatment *in vivo*, mirroring the findings of others that TKI treatment in CML can revert MF (Eliacik et al., 2015). Surprisingly, α-SMA was also expressed by MKs in treated and untreated CML mice, and their numbers were increased in the spleen of CML transgenic mice and elevated in both BM and spleen upon nilotinib treatment. Interestingly, it has been suggested that embryonic CD41^+^ cells from the aorta-gonad-mesonephros expressed mesenchymal markers and displayed myofibroblastic potential and could differentiate into α-SMA-positive cells (Zhou et al., 2012). MKs are found increased in CML (Theologides, 1972; Thielen et al., 2011; Mondet et al., 2015; Hussein et al., 2017) and their numbers positively correlate with the grade of fibrosis in the BM of CML patients (Thiele et al., 2000; Rao et al., 2005).

The impact of this increase of MKs requires further study, not only to address their potential role as a driver of MF in CML, but also to assess whether they might directly support LSC persistence. It has been shown that malignant MKs produce TGF-β, which supports leukemogenic capacity of CML cells (Tanabe et al., 2020), and also, MK-derived expression of TGF-β and CXCL4 is known to maintain HSC quiescence (Bruns et al., 2014; Zhao et al., 2014). Moreover, we and others have previously shown that potent LSCs also reside in the CML spleen (Schemionek et al., 2012; Buhrer et al., 2022) and combined with the splenic increase in MKs, this further supports the rationale to study MK-driven LSC persistence. In line with the observation of Tanabe et al. (2020), where in CML BM-MKs correlated with *BCR::ABL1* copy number after TKI treatment, we could speculate that the persistence of this MK population might represent an obstacle for the success of TFR.

With regard to the role of MSCs in fibrosis development, we observed that MSCs from CML patients increasingly differentiate into adipogenic and possibly osteogenic cell types. MSCs with an adipogenic and osteogenic gene expression pattern have been shown to promote fibrosis in mice (Schepers et al., 2013; Leimkuhler et al., 2021) and a beneficial role of the normal adipo-/osteogenic niche in the maintenance and regulation of normal SC (Omatsu et al., 2010) and LSCs (Schepers et al., 2013) has been shown. The increased adipogenic potential observed in our CML-derived MSCs might suggest a role of adipocytes in the maintenance of LSCs, although the role of marrow adipose tissue in malignant hematopoiesis is still not completely understood. A possible hypothesis is that adipocytes might sustain the survival and proliferation of blasts by supplying them with free fatty acid, similarly to what has been described in AML (Shafat et al., 2017). Moreover, under steady state and metabolic stress conditions, BM adipocytes have been identified as major supplier of stem cell factor, which both malignant and healthy HSC critically depend on (Agarwal et al., 1995; Zhou et al., 2017; Zhang et al., 2019). Furthermore, pre-adipocytic MSCs have been shown to highly express stem cell supporting factors (Matsushita et al., 2020). On the other hand, the presence of mature osteoblasts has been shown to impair Ph^−^ MPN development (Krevvata et al., 2014) revealing that the complexity of the underlying mechanism is hardly understood. MSCs have been previously shown to support LSCs in CML. E.g., it was shown that C-X-C motif chemokine ligand 12 (CXCL12) deletion from mesenchymal progenitors enhanced sensitivity of LSCs to TKI treatment and downregulated mechanisms associated with LSC quiescence (Agarwal et al., 2019). Moreover, BM MSC gene profiling in CML patients at diagnosis and in DMR showed that LSC supporting genes were upregulated despite TKI treatment (Aggoune et al., 2017), further reinforcing that MSCs play a fundamental role in supporting LSC persistence.

When we studied the gene expression profile of CML-MSCs, we found that the signature reflecting fibrosis and hematopoiesis support was altered. In particular, the activation of TGF-β signaling in MSCs might be of interest, since TGF-β is both involved in fibrosis development and in supporting LSC persistence in CML, further tying fibrosis and LSC persistence together (Naka et al., 2010). Furthermore, expression of the gene set MIR431 was downregulated in our analyses. MIR431 has previously been shown to be downregulated in AML and overexpression induced downregulation of TGF-β and EMT markers like fibronectin, α-SMA, and vimentin (Jiang et al., 2018). EMT was likewise significantly regulated as one of the hallmark gene sets tempting to speculate that EndMT (endothelial-to-mesenchymal transition) could potentially be a further source of mesenchymal cells, as previously been suggested in Ph^−^ MPN (Erba et al., 2017).

It is important to mention that the association between persistence of LSC and failure of TFR is still matter of debate. The persistence of LSC in the BM is likely guaranteed by a combination of factors, such as LSC-intrinsic survival pathways, protection provided by the BM niche as well as immune control [Reviewed in (Soverini et al., 2021)]. Importantly, LSC are known to hijack the BM niche, which in turn will nurture malignant cells at the expense of normal HSC. For example, altered levels of pro-inflammatory cytokines or chemokines that favor LSC self-renewal, are found in CML BM even upon TKI-induced remission (Zhang et al., 2012; Zhang et al., 2016). If changes occur in the processes governing the balance between LSC and healthy HSC, LSC might find themselves in a fostering soil for their expansion.

A final link between MF and LSC persistence became evident when we further analyzed our cohort of CML patients who reached the requirements for TKI discontinuation. Specifically, we could observe an association between BM fibrosis and TFR outcome, since the totality of patients that failed TFR had MF at diagnosis.

Nevertheless, it is important to underline that this correlation was not statistically significant (*p* = 0.06) and further studies on larger cohorts are necessary to confirm this association.

Moreover, another important aspect to discuss is that according to the literature, the correlation between the common risk scores and TRF success is not always evident. In some studies, patients with low-to intermediate-risk Sokal score had a significantly higher probability of persisting complete molecular response compared to those with high-risk score (Mahon et al., 2010; Yhim et al., 2012). In our study and in Ross *et al* (Ross et al., 2013), no significant correlation was observed.

In conclusion, in our hypothesis the BM fibrosis in CML patients likely recedes upon TKI treatment, to a macroscopically undetectable level. Nevertheless, such a process fundamentally impacts on the BM niche and one could hypothesize that specific changes might remain on a molecular level, which could drive LSC re-expansion. However, this needs further comparative studies, ideally at the single cell level, of the LSC niche in patients who either relapse after discontinuation of the TKI or who do not.

However, as the grading of MF seems to vary significantly depending on the individual assessment, another, possibly technology-aided and centralized approach to grade fibrosis in the BM would help to reduce the variations seen in different studies (Gralnick et al., 1971; Kvasnicka et al., 2001; Buesche et al., 2003; Hamid et al., 2019). One major limitation of our study is that in our cohort, only a small number of patients in TFR could be included. Therefore, larger studies involving centralized MF evaluation are certainly needed for more robust conclusion on the impact of MF on TFR outcome. However, our data suggest that this might bear the potential to significantly improve the prediction of TFR outcome and moreover, suggest follow-up studies on the underlying mechanisms of fibrotic transformation in CML in the context of LSC persistence.

## Data Availability

The datasets presented in this study can be found in online repositories. The names of the repository/repositories and accession number(s) can be found below: https://www.ncbi.nlm.nih.gov/geo/, GSE230534.
