# Supplementary figures and images for: Myelofibrosis at diagnosis is associated with the failure of treatment-free remission in CML patients
Source: Front Pharmacol. 2023 Jul 4;14:1212392. doi: 10.3389/fphar.2023.1212392 (PMC10352620; doi:10.3389/fphar.2023.1212392)

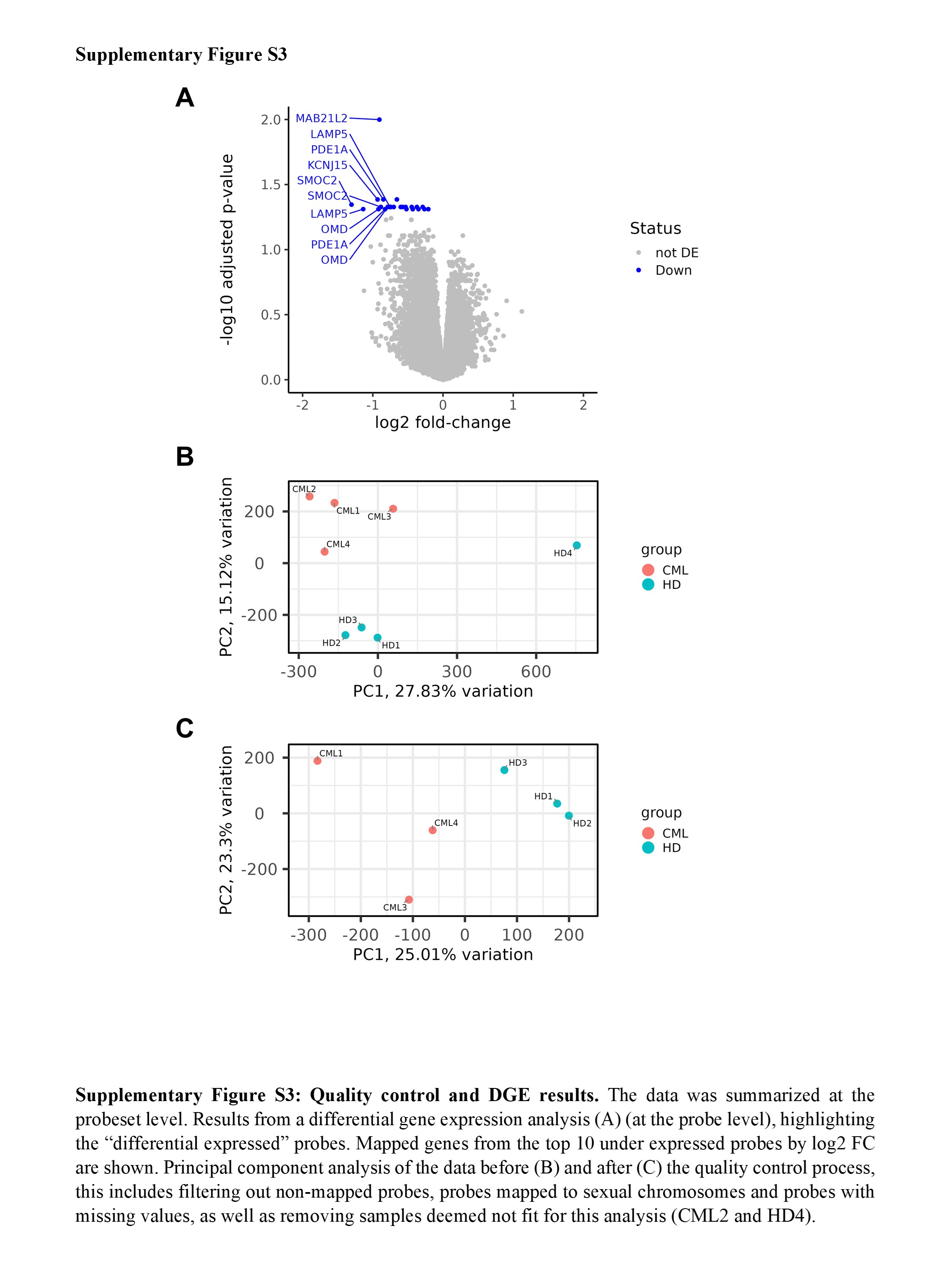

Supplement: Supplementary file 1 [file Image3.jpg]

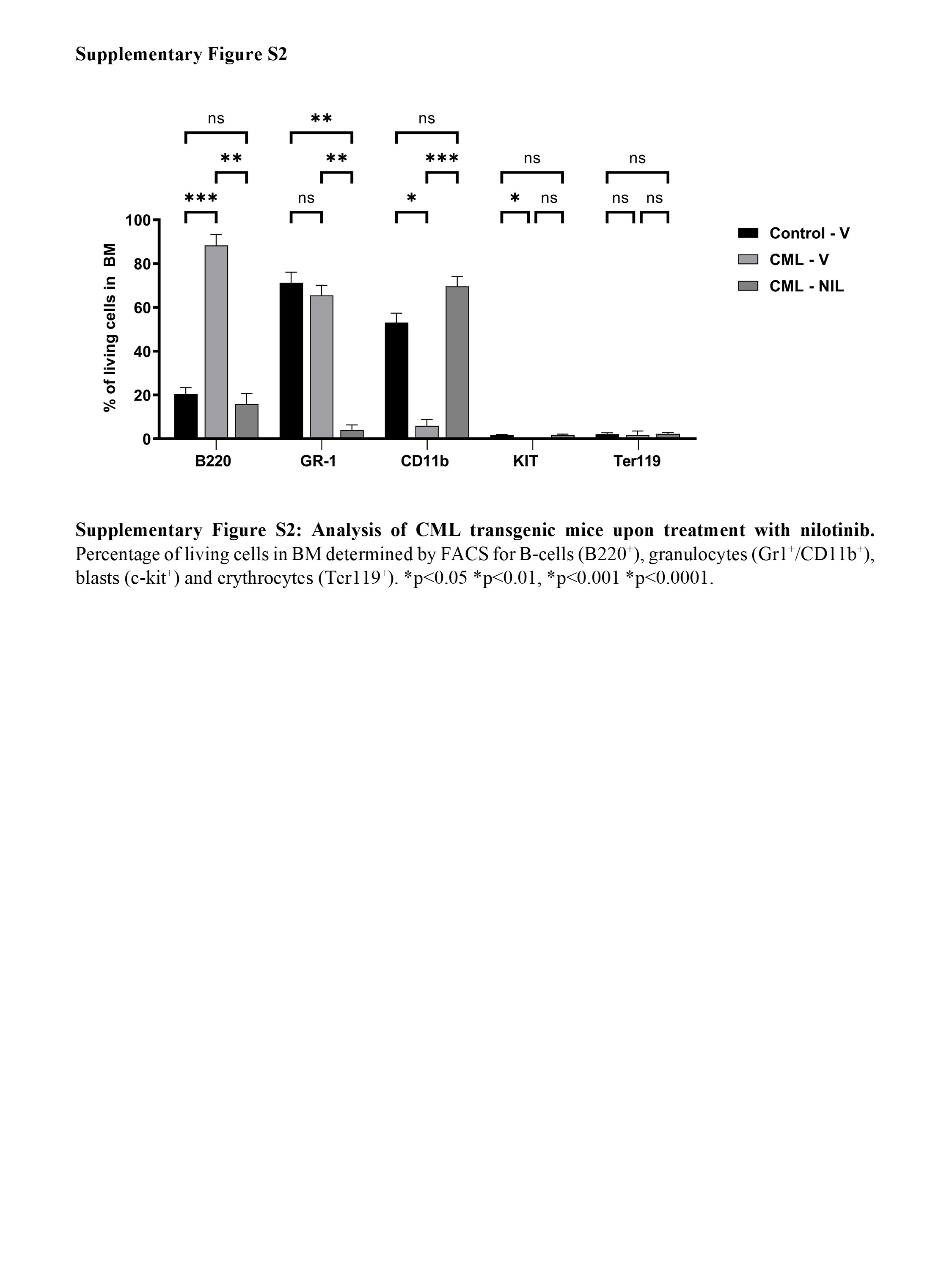

Supplement: Supplementary file 2 [file Image2.jpg]

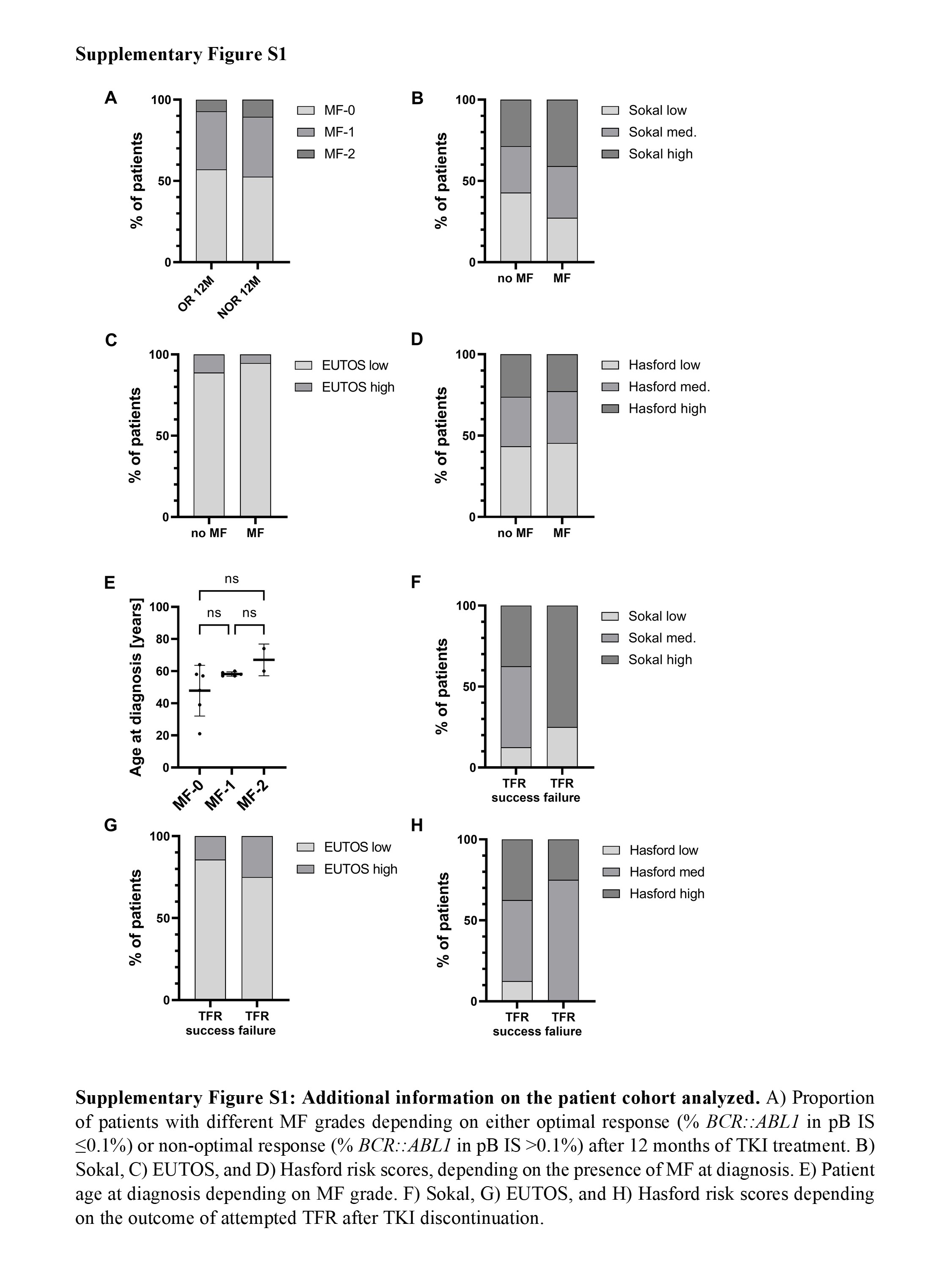

Supplement: Supplementary file 4 [file Image1.jpg]
