# Supplementary material for: Myelofibrosis at diagnosis is associated with the failure of treatment-free remission in CML patients
Source: Front Pharmacol. 2023 Jul 4;14:1212392. doi: 10.3389/fphar.2023.1212392 (PMC10352620; doi:10.3389/fphar.2023.1212392)
Supplement: Supplementary file 3 [file Table1.pdf]

Supplementary Table S1: Risk scores and myelofibrosis follow-up of patients that discontinued TKI.

No.	Spleen [cm]	Sokal	EUTOS	Hasford	MF grade at diagnosis	MF grade at other timepoints
1	14	low	low	med	0	5 months after diagnosis: MF-0
2	23	high	-	high	0	3, 6 and 12 months after diagnosis: MF-0
3	21	med	low	low	0	6 months, 3 and 4.5 years after diagnosis: MF-0; 5.5 years after diagnosis: MF-1
4	19.2	med	low	med	0	no further MF information
5	9.2	med	low	med	0	no further MF information
6	25	high	high	high	0	no further MF information
7	13	high	low	med	1	3 months after diagnosis: MF-0; 1.5 and 2.5 years after diagnosis: MF-1
8	14.4	med	low	med	1	no further MF information
9	11	low	low	med	1	no further MF information
10	11	high	low	high	1	no further MF information
11	18.2	low	low	low	1	3 years after diagnosis: MF-0
12	16.6	high	low	med	2	no further MF information
13	20	high	high	high	2	no further MF information

No.: number, med: intermediate, MF: myelofibrosis. Spleen lengths, Sokal, EUTOS and Hasford risk scores are given for the timepoint of diagnosis
